# Bleaching Teeth

**Published:** 1865-07

**Authors:** Wm. H. Atkinson


Bleaching Teeth.—*By Wm. H. Atkinson, M. D.:* Read before the Dental Association.—To understand the process of bleaching teeth and to successfully practice it, involves a deeper erudition in philosophy than is common even among professional men. It requires minute anatomical apprehension, no less than insight, of the movements of the small bodies that elaborate the function of nutrition, the process by which tissues are grown and destroyed. We might be completely conversant with these movements as to their relations to demand, supply, appropriation, and distribution, or rejection of the unprepared or incompatible foods presented to them, and the waste material of their own personalities as they become effete, and yet if we omitted the study of their relations to light we should utterly fail of comprehending the principle or power which effects the molecular process we denominate bleaching.

This question can be apprehended only by the comprehension of the laws of optics, the phenomenon of light, and the changes occurring in physiological and pathological nutrition, and the partial and complete solution of the organs we contemplate. Probably no chemical change can take place in a body without the physical relation of its atoms also suffering change. But what degree of derangement is necessary to vary the absorbing, reflecting and refracting power of the body to make it apparent to the eye, has not been determined. In fact, the ability to detect slight differences of shade and color varies so infinitely in the different degrees of natural power and education of the faculty, that there can hardly be found two individuals to agree upon a standard that shall be satisfactory in its details. Some detect shades well who are sharp in the apprehension of color; while othrrs, who detect readily strong contrasts in color, can hardly distinguish shades when nearly allied. The nice perception is pained at the presentation of degrees of difference that would pass quite unnoticed by the dull or undeveloped perception of differentiations, and hence the everlasting jangle among dentists and patients on these subjects.

Now it is not pretended that any one knows the solutions to the questions that arise when we contemplate the subject of bleaching the substances which are usually subjected to this process for the production of textile fabrics, much less that we understand the less definite and more dificultly managed subject of bleaching teeth in the mouth. But because we have not yet exhausted the detail of this matter, it is no reason why we should not take the more pains to peer into the dark abyss before us, until we shall have developed increased powers of perception and understanding respecting the occult laws and processes whose works are displayed in the changes producing the evil that bleaching is invoked to remedy. Most of our processes for bleaching are, as yet, purely empirical or experimental, as are most of the efforts at healing the other ills to which we have fallen heirs.

That which constitutes transparency is doubtless unitary relations of arrangement of the molecules of a body. This is proven in the examples of coloration and decoloration displayed in the demonstrations of chemical teachers, who mix various solutions of diverse affinities to produce colors, and those of mutual affinities to reduce these again to colorless, transparent, or opaque solutions, just as unity or diversity is attained in molecular movements.

Any agent strong enough in solvent power to completely break up the molecular arrangement of the particles of a body is capable of producing its *clear* solution. Adventitious and opposing affinities and movements interfere in the degree of their presence. Hence we are quite at a loss as to the particular molecular changes which accompany the degrees of refraction, reflection, and absorption that accompany the decolorations amenable to bleaching.

Teeth are liable to discoloration, which renders them unsightly rather than useless. Various causes contribute to this vexatious result. It arises principally from death and disintegration of the pulp, spontaneously or adventitiously produced. Pulps may die and be absorbed without great change in the color of the crowns to which they belonged. But, generally, when sudden death of a pulp takes place, it decomposes, infiltrating the structure of the dentine with the coloring matter of the blood, turning the tooth pink, gray, brown, or blue.

The younger the patient the more disastrous will be the conditions which cause discoloration of the teeth. The chief reason of this is the porosity of the dentine being so much greater than in adults and persons advanced in life, whose tubuli become consolidated, or at least much attenuated, reducing the calibre capable of containing matter, the changes in which, produce discoloration. I have discovered one form of discoloration that renders the teeth subject thereto even more indestructible than other teeth in the same mouth which are regarded healthy. I refer to that particular calcific infiltration of the tubules with limesalts that gives the tooth a deeper shade of yellow than even the canines of dense structure so common in well organized teeth.

There is one form of death of the pulp which produces so little change in color of the tooth that it may be present for a long time without detection. I refer to the death by benign suppuration, leaving the converted pulp "in situ," which prevents molecular change in the contents of the tubules to the degree necessary to effect the light-pencil sufficiently to cause the detection of the state, short of the closest observation. It is fortunate for us that this is not destructive to the strength of the tooth until further change takes place in the pus column, generating acids or gases, whose actions are soon apparent through change in color.

It is profitable to inquire, what have our empirical observations and efforts taught us upon this subject? They have taught us by experience that the great desideratum concerning the color of "devitalized teeth" (so called) is, imperviously closing of the open ends of the tubules. A thing, by the way, difficult to do, and scarcely ever attained by any but the most patient and most painstaking members of our body. But if we set about it with this knowledge beforehand, we are more likely to see our efforts successful. No mere haphazard procedure can be uniform in its results. First comprehend the principle and then adopt modes of procedure thereto, and the best will soon stand revealed to our vision.

The best results thus far attained have succeeded the following procedure, viz.: Cutting away the discolored dentine, so far as possible, without too much compromising the safety of the enamel, then carefully fill the root or roots nearly to the nerve chamber proper, in the tooth. After hermetically stopping the canal thus far, proceed to introduce chlorine in some form. Some prefer the gas, freshly generated, conducted directly into the cavity by means of a small glass tube, drawn to a fine point to enable the operator to direct the current as it flows immediately against and into the open ends of the tubes of the discolored dentine. The only serious objection to this method is the liability to produce unpleasant congestion and trouble of the mucous membrane of the air passages. Some use common chloride of lime, introducing it in a wet state, and changing it once in five or ten minutes, until the desired bleaching is obtained. Others introduce precipitated chalk made into a mortar, with Labarraque's solution of the chloride of soda, changing this often until the tooth is sufficiently bleached.

In case any or all these methods fail to produce the desired result, dismiss your patient for a day or two, advising him to keep the cavity open to the air and free from food and all foreign substances, closing it with wax, cotton, or paper, during eating; remove it, and rinse freely with a solution of soda immediately after. If these steps be faithfully taken, the worst cases will yield and be ready for satisfactory filling in less than one week.—*Dental Cosmos.*

